# Supplementary material for: Whole-Blood Longitudinal Molecular Profiling Maps the Road of Graft Versus Host Disease (GVHD)
Source: Cancers (Basel). 2025 Feb 26;17(5):802. doi: 10.3390/cancers17050802 (PMC11899482; doi:10.3390/cancers17050802)
Supplement: Supplementary file 1 [file cancers-17-00802-s001.zip › Figure S2.pptx]

## Slide 1
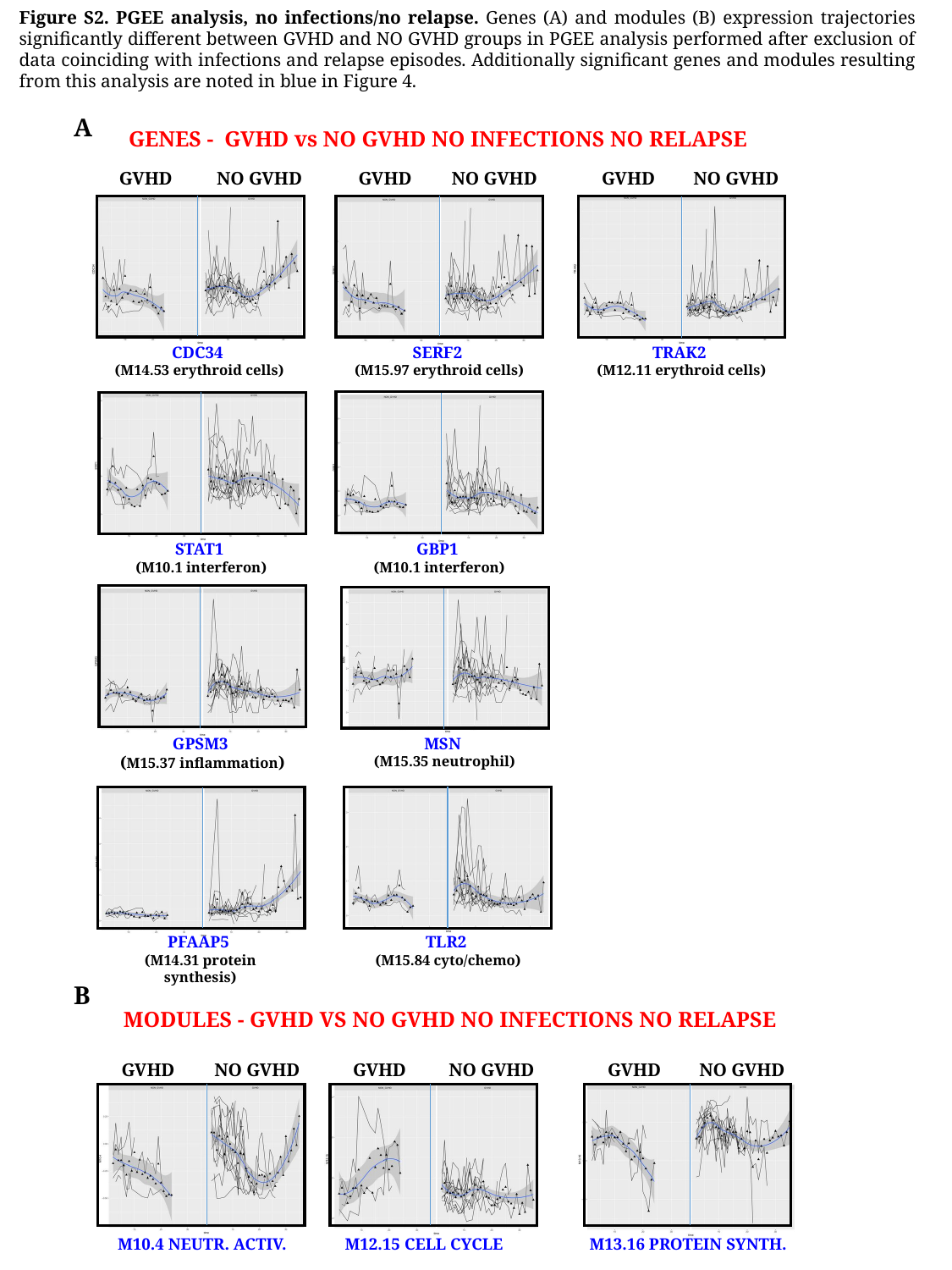

Figure S2. PGEE analysis, no infections/no relapse. Genes (A) and modules (B) expression trajectories significantly different between GVHD and NO GVHD groups in PGEE analysis performed after exclusion of data coinciding with infections and relapse episodes. Additionally significant genes and modules resulting from this analysis are noted in blue in Figure 4.
A
GENES - GVHD vs NO GVHD NO INFECTIONS NO RELAPSE
GVHD
NO GVHD
GVHD
NO GVHD
GVHD
NO GVHD
CDC34
(M14.53 erythroid cells)
SERF2
(M15.97 erythroid cells)
TRAK2
(M12.11 erythroid cells)
STAT1
(M10.1 interferon)
GBP1
(M10.1 interferon)
GPSM3
(M15.37 inflammation)
MSN
(M15.35 neutrophil)
PFAAP5
(M14.31 protein synthesis)
TLR2
(M15.84 cyto/chemo)
B
MODULES - GVHD VS NO GVHD NO INFECTIONS NO RELAPSE
GVHD
NO GVHD
GVHD
NO GVHD
GVHD
NO GVHD
M10.4 NEUTR. ACTIV.
M12.15 CELL CYCLE
M13.16 PROTEIN SYNTH.
