# Supplementary material for: Whole-Blood Longitudinal Molecular Profiling Maps the Road of Graft Versus Host Disease (GVHD)
Source: Cancers (Basel). 2025 Feb 26;17(5):802. doi: 10.3390/cancers17050802 (PMC11899482; doi:10.3390/cancers17050802)
Supplement: Supplementary file 1 [file cancers-17-00802-s001.zip › FigureS1.pdf]

Figure S1. List of Genes, Modules and Aggregates. A complete list of Genes, Modules (combination of 4 genes) and Aggregates (combinations of 2 to 42 modules) used for the analysis is provided.

Aggregate	Module	Function	symbol
A28	M10.1	Interferon	GBP1
A28	M10.1	Interferon	IFI35
A28	M10.1	Interferon	STAT1
A28	M10.1	Interferon	ZBP1
A9	M10.2	Protein synthesis	HBB
A9	M10.2	Protein synthesis	LAIR1
A9	M10.2	Protein synthesis	OAZ1
A9	M10.2	Protein synthesis	RPS12
A38	M10.4	Neutrophil activation	CEACAM6
A38	M10.4	Neutrophil activation	CEACAM8
A38	M10.4	Neutrophil activation	DEFA4
A38	M10.4	Neutrophil activation	ELANE
A37	M12.11	Erythroid cells	RAD23A
A37	M12.11	Erythroid cells	RPIA
A37	M12.11	Erythroid cells	SIAH2
A37	M12.11	Erythroid cells	TRAK2
A27	M12.15	Plasma cells	CD38
A27	M12.15	Plasma cells	MGC29506
A27	M12.15	Plasma cells	TNFRSF17
A27	M12.15	Plasma cells	TYMS
A26	M12.2	Monocytes	ALDH2
A26	M12.2	Monocytes	CEBPA
A26	M12.2	Monocytes	EMILIN2
A26	M12.2	Monocytes	KYNU
A1	M12.3	Cell cycle	ELP3
A1	M12.3	Cell cycle	LANCL1
A1	M12.3	Cell cycle	NUP160
A1	M12.3	Cell cycle	TTC4
A2	M12.4	Gene transcription	C19ORF53
A2	M12.4	Gene transcription	CCDC12
A2	M12.4	Gene transcription	E4F1
A2	M12.4	Gene transcription	NDUFA8
A3	M12.5	Protein modification	CCDC16
A3	M12.5	Protein modification	INTS10
A3	M12.5	Protein modification	RPS6KB1
A3	M12.5	Protein modification	ZFYVE20
A1	M12.8	B cells	COBLL1
A1	M12.8	B cells	HLA-DOA
A1	M12.8	B cells	HLA-DOB
A1	M12.8	B cells	VPREB3
A35	M13.12	Inflammation	C5ORF32
A35	M13.12	Inflammation	HK3
A35	M13.12	Inflammation	PGS1
A35	M13.12	Inflammation	RGL4
A35	M13.16	Cytokines/chemokines	ALPK1
A35	M13.16	Cytokines/chemokines	GK
A35	M13.16	Cytokines/chemokines	KCNJ2
A35	M13.16	Cytokines/chemokines	LRG1
A28	M13.17	Interferon	IFI16
A28	M13.17	Interferon	NT5C3
A28	M13.17	Interferon	SAMD9
A28	M13.17	Interferon	SP110
A1	M13.18	Lymphocytes	GOT1
A1	M13.18	Lymphocytes	RPP40
A1	M13.18	Lymphocytes	TRIAP1
A1	M13.18	Lymphocytes	ZNF17
A30	M13.19	Protein phosphorylation	CEP350
A30	M13.19	Protein phosphorylation	MAP3K5
A30	M13.19	Protein phosphorylation	PIK3CG
A30	M13.19	Protein phosphorylation	PPTC7
A2	M13.21	Cytotoxic lymphocytes	CTSW
A2	M13.21	Cytotoxic lymphocytes	FLJ20699
A2	M13.21	Cytotoxic lymphocytes	PYHIN1
A2	M13.21	Cytotoxic lymphocytes	SAMD3
A1	M13.27	Lymphocytes	CD6
A1	M13.27	Lymphocytes	CD96
A1	M13.27	Lymphocytes	GIMAP5
A1	M13.27	Lymphocytes	SKAP1
A24	M14.30	Oxidative phosphorylati	C11ORF48
A24	M14.30	Oxidative phosphorylati	DDT
A24	M14.30	Oxidative phosphorylati	NDUFA11
A24	M14.30	Oxidative phosphorylati	RPS21
A3	M14.31	Cell cycle	ANAPC4
A3	M14.31	Cell cycle	AP3M2
A3	M14.31	Cell cycle	NAT9
A3	M14.31	Cell cycle	PFAAP5
A31	M14.48	Inflammation	CTSS
A31	M14.48	Inflammation	DPEP2
A31	M14.48	Inflammation	FCGRT
A31	M14.48	Inflammation	NUP214
A37	M14.53	Erythroid cells	CDC34
A37	M14.53	Erythroid cells	CHPT1
A37	M14.53	Erythroid cells	IGF2BP2
A37	M14.53	Erythroid cells	RBM38
A35	M14.65	Monocytes	CLIC1
A35	M14.65	Monocytes	CYBA
A35	M14.65	Monocytes	EFHD2
A35	M14.65	Monocytes	IFNGR2

Aggregate	Module	Function	symbol
A32	M14.67	Gene transcription	C18ORF32
A32	M14.67	Gene transcription	EAF1
A32	M14.67	Gene transcription	FLI1
A32	M14.67	Gene transcription	SAP30L
A33	M14.76	Leukocyte activation	CD93
A33	M14.76	Leukocyte activation	EIF2C4
A33	M14.76	Leukocyte activation	FAM8A1
A33	M14.76	Leukocyte activation	PECAM1
A33	M14.82	Cytokines/chemokines	CCDC89
A33	M14.82	Cytokines/chemokines	MED13L
A33	M14.82	Cytokines/chemokines	NANS
A33	M14.82	Cytokines/chemokines	SPATA2L
A10	M15.102	Prostanoids	GPR44
A10	M15.102	Prostanoids	IL5RA
A10	M15.102	Prostanoids	OLIG2
A10	M15.102	Prostanoids	PRSS33
A12	M15.125	TBD	HUWE1
A12	M15.125	TBD	PSMD5
A12	M15.125	TBD	SIPA1L3
A12	M15.125	TBD	TEX261
A28	M15.127	Interferon	CMPK2
A28	M15.127	Interferon	IFI27
A28	M15.127	Interferon	IFITM3
A28	M15.127	Interferon	SPATS2L
A3	M15.16	Antigen presentation	ALKBH2
A3	M15.16	Antigen presentation	AMER1
A3	M15.16	Antigen presentation	CYCS
A3	M15.16	Antigen presentation	IL23A
A33	M15.35	Neutrophils	CAP1
A33	M15.35	Neutrophils	MSN
A33	M15.35	Neutrophils	RAC2
A33	M15.35	Neutrophils	TXNIP
A35	M15.37	Inflammation	GPSM3
A35	M15.37	Inflammation	NDUFB3
A35	M15.37	Inflammation	NT5C2
A35	M15.37	Inflammation	SH3GLB1
A8	M15.39	TBD	AP1M1
A8	M15.39	TBD	IKBKG
A8	M15.39	TBD	MGC3731
A8	M15.39	TBD	RAB40C
A1	M15.4	Lymphocytes	FAM44B
A1	M15.4	Lymphocytes	RAN
A1	M15.4	Lymphocytes	SLC35A3
A1	M15.4	Lymphocytes	ZNF7
A6	M15.40	Gene transcription	C1ORF35
A6	M15.40	Gene transcription	DNAJC27
A6	M15.40	Gene transcription	PIGK
A6	M15.40	Gene transcription	TMEM170A
A6	M15.42	Gene transcription	GOPC
A6	M15.42	Gene transcription	HIVEP2
A6	M15.42	Gene transcription	SNW1
A6	M15.42	Gene transcription	TMEM199
A32	M15.47	Antigen presentation	IK
A32	M15.47	Antigen presentation	INPP5D
A32	M15.47	Antigen presentation	SASH3
A32	M15.47	Antigen presentation	UBE2J1
A4	M15.49	Cell death	CCNK
A4	M15.49	Cell death	PPP3CB
A4	M15.49	Cell death	RCOR3
A4	M15.49	Cell death	UBL3
A5	M15.5	Protein modification	COPB1
A5	M15.5	Protein modification	GNG2
A5	M15.5	Protein modification	SMEK2
A5	M15.5	Protein modification	TAF7
A25	M15.55	Protein phosphorylation	DMXL2
A25	M15.55	Protein phosphorylation	HERC3
A25	M15.55	Protein phosphorylation	KIF5B
A25	M15.55	Protein phosphorylation	NLRC5
A31	M15.58	Monocytes	CDC42EP2
A31	M15.58	Monocytes	EMR3
A31	M15.58	Monocytes	KIAA1324
A31	M15.58	Monocytes	PTGS2
A7	M15.61	Monocytes	ANKRD57
A7	M15.61	Monocytes	KCNMB1
A7	M15.61	Monocytes	SLC27A1
A7	M15.61	Monocytes	ZFHX3
A28	M15.64	Interferon	CCR1
A28	M15.64	Interferon	LBA1
A28	M15.64	Interferon	TRIM38
A28	M15.64	Interferon	TRIM56
A11	M15.67	TBD	C19ORF56
A11	M15.67	TBD	C1ORF144
A11	M15.67	TBD	HMG20B
A11	M15.67	TBD	SPSB3
A26	M15.7	Monocytes	ADCY7
A26	M15.7	Monocytes	FCHSD2
A26	M15.7	Monocytes	PAF1
A26	M15.7	Monocytes	TTC1

Aggregate	Module	Function	symbol
A35	M15.84	Cytokines/chemokines	OSM
A35	M15.84	Cytokines/chemokines	SLC2A14
A35	M15.84	Cytokines/chemokines	ST3GAL4
A35	M15.84	Cytokines/chemokines	TLR2
A28	M15.86	Interferon	GALM
A28	M15.86	Interferon	KIAA1618
A28	M15.86	Interferon	MOV10
A28	M15.86	Interferon	TIMM10
A36	M15.97	Erythroid cells	C12ORF10
A36	M15.97	Erythroid cells	ROGDI
A36	M15.97	Erythroid cells	SERF2
A36	M15.97	Erythroid cells	SH3GLB2
A13	M16.108	TBD	ASRGL1
A13	M16.108	TBD	FMO5
A13	M16.108	TBD	PI4K2A
A13	M16.108	TBD	SPIN3
A15	M16.12	B cells	CLIC5
A15	M16.12	B cells	METTL14
A15	M16.12	B cells	SH3BP2
A15	M16.12	B cells	ZNF292
A5	M16.18	TBD	C11ORF31
A5	M16.18	TBD	DISP1
A5	M16.18	TBD	SALL2
A5	M16.18	TBD	ZNF543
A6	M16.3	T cells	CLN5
A6	M16.3	T cells	RASA3
A6	M16.3	T cells	SEC22A
A6	M16.3	T cells	ZNF212
A8	M16.30	Complement	CPSF6
A8	M16.30	Complement	MTHFD1
A8	M16.30	Complement	PRR11
A8	M16.30	Complement	TMEM237
A33	M16.44	Protein synthesis	ABHD5
A33	M16.44	Protein synthesis	ACTN4
A33	M16.44	Protein synthesis	ACTR10
A33	M16.44	Protein synthesis	PTAFR
A18	M16.47	TNF	CASC1
A18	M16.47	TNF	DCAF4L1
A18	M16.47	TNF	PPIF
A18	M16.47	TNF	RAB13
A20	M16.52	Lymphocyte	C14ORF11[illegible]
A20	M16.52	Lymphocyte	EPHX4
A20	M16.52	Lymphocyte	LHFPL1
A20	M16.52	Lymphocyte	PLK3
A8	M16.6	Monocytes	C18ORF19
A8	M16.6	Monocytes	C5ORF22
A8	M16.6	Monocytes	PRMT7
A8	M16.6	Monocytes	TRUB2
A31	M16.64	Platelet/Prostaglandin	C5ORF62
A31	M16.64	Platelet/Prostaglandin	DDEF2
A31	M16.64	Platelet/Prostaglandin	MAP1A
A31	M16.64	Platelet/Prostaglandin	PNMA1
A15	M16.66	TBD	KRI1
A15	M16.66	TBD	MC1R
A15	M16.66	TBD	SCNN1D
A15	M16.66	TBD	ZNF248
A4	M16.77	Antigen presentation	ACOT4
A4	M16.77	Antigen presentation	MAP3K12
A4	M16.77	Antigen presentation	NCR3LG1
A4	M16.77	Antigen presentation	THG1L
A1	M16.78	Lymphocytes	CD1C
A1	M16.78	Lymphocytes	CDR2
A1	M16.78	Lymphocytes	FAM216A
A1	M16.78	Lymphocytes	PPAPDC1[illegible]
A33	M16.80	Cytokines/chemokines	BAGE3
A33	M16.80	Cytokines/chemokines	BTNL9
A33	M16.80	Cytokines/chemokines	KIAA0319
A33	M16.80	Cytokines/chemokines	PLB1
A29	M3.1	Cell cycle	HSPC268
A29	M3.1	Cell cycle	NUBPL
A29	M3.1	Cell cycle	PIP5K2B
A29	M3.1	Cell cycle	ZNF786
A29	M8.1	TBD	DNAJC28
A29	M8.1	TBD	ERAP2
A29	M8.1	TBD	IL18
A29	M8.1	TBD	MBD4
A34	M8.2	Prostanoids	CTDSPL
A34	M8.2	Prostanoids	SH3BGRL2
A34	M8.2	Prostanoids	TSPAN33
A34	M8.2	Prostanoids	TSPAN9
A28	M8.3	Type 1 Interferon	IFI44
A28	M8.3	Type 1 Interferon	ISG15
A28	M8.3	Type 1 Interferon	LY6E
A28	M8.3	Type 1 Interferon	XAF1
A2	M9.1	Cytotoxic lymphocytes	EOMES
A2	M9.1	Cytotoxic lymphocytes	KLRF1
A2	M9.1	Cytotoxic lymphocytes	PRF1
A2	M9.1	Cytotoxic lymphocytes	TGFBR3
